# Cognitive impairment in patients treated for stroke in Chad

**DOI:** 10.1002/alz70857_104406

**Published:** 2025-12-25

**Authors:** Foksouna Sakadi, Nderbe Melom Christelle, Allaissem Doumdongar, Kamis Dakisia, Alfred Kongnyu Njamnshi

**Affiliations:** ^1^ Department of Neurology, Reference Hospital, Ndjamena, NDdjamena, Ndjamena, Chad; ^2^ Nationale Reference Teaching Hospital, Ndjamena, Chad; ^3^ National Reference Teaching Hospital, N'Djamena, Chad; ^4^ National Refrence Teaching Hospital, N'Djamena, Chad; ^5^ Brain Research Africa Initiative, Yaounde, Centre, Cameroon; ^6^ The University of Yaoundé I, Yaoundé, Cameroon, Cameroon

## Abstract

**Background:**

Stroke is a serious public health problem and neurological condition with a huge socio‐economic burden related to its complications. However, cognitive complications of stroke have not been studied in Chad. Therefore, this study aimed at describing cognitive impairment in patients followed up for stroke with the goal of improving management.

**Method:**

This was a cross‐sectional descriptive multicenter study conducted over an 8‐month period (30^th^ November to 31^st^ July 2023). The study took place in three referral hospitals in Ndjamena: CHURN, CHU‐R and HATC. All patients admitted for stroke and diagnosed with cognitive impairment according to VASCOG criteria were included. The variables studied were: sociodemographic, anamnestic, clinical and paraclinical. The MMSE, the Albert test, the Rey figure, the ISDC test were used to assess cognitive impairment while the Goldberg Anxiety and Depression (GADS) scale assessed level of anxiety/depression and the Barthel scale provided information on participant's activities of daily living.

**Result:**

During the study period, we enrolled 24 patients (58.3% males) and 18.18% of total sample had cognitive impairment. The mean age of the patients was 58.17 ± 11.99 years, (range: 48 – 89). All our patients had consulted for memory problems and on examination, 20 (%) had motor deficits and 12 (%) had language disorders. The MMSE ranged from [0‐17] in 14 patients (%) to [18‐23] in 6 patients (%). The Albert test revealed perceptual neglect in 16 (%) patients. The Barthel scale found 41.7% of patients to be totally dependent, and the GADS revealed moderate depression in 41% of patients.

**Conclusion:**

This study showed that cognitive impairment is frequently associated with stroke and aggravates patient dependence. There is need for more in‐depth with a more comprehensive neuropsychological batteries and imaging studies.

